# Supplementary material for: Comprehensive Analysis of Oncogenic Somatic Alterations of Mismatch Repair Gene in Breast Cancer Patients
Source: Bioengineering (Basel). 2025 Apr 18;12(4):426. doi: 10.3390/bioengineering12040426 (PMC12025084; doi:10.3390/bioengineering12040426)

Online – Supplementary Material

Comprehensive analysis of oncogenic somatic alterations of mismatch repair gene in breast cancer patients

Yin Yan[1†], Yang Wang[2†], Junjie Tang[3†], Xiaoran Liu[1], Jichuan Wang[4], Guohong Song[1]* and Huiping Li[1]*

Contents

Supplementary Tables.

Table S1. Prevalence of pathogenic somatic mutations in MMR genes in various public datasets.

Table S2. Forty-four breast cancers with pathogenic somatic mutations in MMR genes identified from various public datasets.

Table S3. Comparison of the effects of various MMR mutation types on MMR function and immunogenicity in breast cancer.

Table S4. Comparison of MMR function and immunogenicity between biallelic and monoallelic inactivation of MMR genes in breast cancers.

Table S5. Comparison of alteration frequencies of driver genes between MMR-altered and MMR-wildtype breast cancers. (provided as a separate Excel file)

Supplementary Figures.

Figure S1. The positive control (PC) and negative control (NC) for MMR protein immunohistochemistry assays.

Table S1. Prevalence of pathogenic somatic mutations in MMR genes in various public datasets.

	No. of patients	Methods of sequencing	MMR-altered (rate)
**In total**	**3667**	—	**44 (1.20%)**
brca_cptac_2020	122	WES; bulk RNA-seq	3 (2.46%)
brca_igr_2015	216	WES;	5 (2.31%)
brca_mapk_hp_msk_2021	68	Panel	2 (2.94%)
breast_ink4_msk_2021	451	Panel	5 (1.11%)
breast_msk_2018	1756	Panel	10 (0.57%)
TCGA_brca	1054	WES; bulk RNA-seq	19 (1.80%)

Table S2. Forty-four breast cancers with pathogenic somatic mutations in MMR genes identified from various public datasets.

Study ID	Sample ID	MMR somatic	Age (y)	Histology	Molecular subtype	Stage	TMB (/Mb)	Neoantigen (/Mb)	MSI status	Biallelic Inactivation	MMRDetect class
TCGA_brca	TCGA-A2-A04U-01	MSH2-SLC3A1 Fusion	47	IDC	TNBC	T2N0M0	1.6	2.6	MSS	Neg	Neg
TCGA_brca	TCGA-A2-A1FZ-01	MLH1 HOMDEL	63	IDC	HR+HER2-	T2N0M0	1.0	0.4	MSS	Pos	Neg
TCGA_brca	TCGA-A8-A07R-01	MSH2 HOMDEL; MSH6 HOMDEL	80	IDC	HER2+	T2N3M0	12.4	NA	MSS	Pos	Neg
TCGA_brca	TCGA-AC-A23H-01	MSH6 p.E463*	90	IDC	HER2+	T2M0	128.4	131.6	MSS	Neg	Neg
TCGA_brca	TCGA-AC-A6IW-01	MSH2 p.X879_splice	73	IDC	TNBC	NA	4.4	13.4	MSS	Neg	Neg
TCGA_brca	TCGA-AN-A046-01	MSH6 p.R922*	68	NA	HR+HER2-	N0M0	142.4	158.8	MSS	Pos	Neg
TCGA_brca	TCGA-AN-A0AK-01	MLH1 X347_splice; MSH6 p.F1088Sfs	76	IDC	HER2+	T2N0M0	35.1	24.2	MSI-H	Pos	Pos
TCGA_brca	TCGA-AO-A128-01	MSH6 p.F1088Pfs	61	IDC	TNBC	T2N0M0	28.7	27.6	MSS	Neg	Pos
TCGA_brca	TCGA-B6-A0IK-01	PMS2 p.R134*	63	IDC	TNBC	T4N1M0	4.0	9.6	MSS	Neg	Neg
TCGA_brca	TCGA-BH-A0HA-01	MLH1 p.E102*	31	IDC	HR+HER2-	T1N0M0	8.6	14.1	MSI-H	Pos	Pos
TCGA_brca	TCGA-BH-A18G-01	MLH1 HOMDEL	81	IDC	TNBC	T1N0M0	37.9	51.8	MSI-H	Pos	Pos
TCGA_brca	TCGA-BH-A18L-01	MSH2-TMEM247 Fusion	50	IDC	HR+HER2-	T3N1M0	1.1	1.8	MSS	Neg	NA
TCGA_brca	TCGA-BH-A1F6-01	MSH6 p.K888Sfs	51	IDC	TNBC	T4N2	1.7	2.5	MSS	Neg	Neg

TCGA_brca	TCGA-D8-A1XK-01	MLH1 p.A111P	55	IDC	TNBC	T2N1M0	29.6	22.7	MSI-H	Neg	Pos
TCGA_brca	TCGA-D8-A1Y1-01	MSH2 p.R621*	80	IDC	HR+HER2-	T3N1M0	6.8	8.0	MSI-H	Pos	Neg
TCGA_brca	TCGA-E2-A1LH-01	MLH1 HOMDEL	59	IDC	TNBC	T1N0M0	4.6	6.9	MSS	Pos	Neg
TCGA_brca	TCGA-EW-A2FV-01	PMS2 p.A129Rfs*7	39	NA	HR+HER2-	T3N3M0	0.7	0.6	MSS	Neg	Neg
TCGA_brca	TCGA-GM-A3XL-01	WARS-MSH6 Fusion	49	IDC	TNBC	T2N0M0	2.6	3.5	MSS	Neg	Neg
TCGA_brca	TCGA-LL-A5YP-01	PMS2 HOMDEL	49	IDC	HR+HER2-	T2N1M0	2.1	7.1	MSS	Pos	NA
breast_msk_2018	P-0000138-T01-IM3	MSH2 p.E853*	46	mix	HR+HER2-	M1	0.8	NA	NA	Neg	NA
breast_msk_2018	P-0000201-T01-IM3	MSH2 p.S269*	55	ILC	HR+HER2-	T2N1M1	0.4	NA	NA	Pos	NA
breast_msk_2018	P-0000247-T01-IM3	MSH6 p.S154*	42	IDC	HR+HER2-	T2N1M1	0.3	NA	NA	Neg	NA
breast_msk_2018	P-0000517-T01-IM3	MSH6 p.L655*	48	IDC	TNBC	T1N0M1	0.4	NA	NA	Neg	NA
breast_msk_2018	P-0003727-T01-IM5	PMS2 HOMDEL	49	IDC	TNBC	T1N0M1	0.0	NA	NA	Pos	NA
breast_msk_2018	P-0004555-T01-IM5	MLH1 p.E414*; p.E439*	49	ILC	HR+HER2-	T3N2M1	1.0	NA	NA	Neg	NA
breast_msk_2018	P-0010235-T01-IM5	PMS2 p.E77*	53	IDC	HR+HER2-	T1N2M1	0.3	NA	NA	Neg	NA
breast_msk_2018	P-0014802-T01-IM6	MLH1 p.X702_splice	69	IDC	HR+HER2-	T1N0M0	0.1	NA	NA	Neg	NA
breast_msk_2018	P-0016773-T01-IM6	MSH6 p.S580*	77	IDC	HR+HER2-	T1N0M0	14.8	NA	NA	Pos	NA
breast_msk_2018	P-0018675-T01-IM6	MLH1 p.X102_splice	73	mix	HR+HER2-	T2N0M0	0.4	NA	NA	Pos	NA
breast_ink4_msk_2021	P-0015138-T01-IM6	MSH2 p.E728Vfs	NA	NA	NA	NA	6.1	NA	MSS	Neg	NA

breast_ink4_msk_2021	P-0018939-T01-IM6	MSH2 p.S676*	NA	NA	NA	M1	11.2	NA	MSS	Neg	NA
breast_ink4_msk_2021	P-0020627-T01-IM6	PMS2 p.S128Tfs*10	NA	NA	NA	M1	15.6	NA	MSS	Neg	NA
brca_mapk_hp_msk_2021	P-0026484-T01-IM6	MLH1 p.X702_splice	72	IDC	NA	NA	21.6	NA	MSI-H	Pos	NA
brca_mapk_hp_msk_2021	P-0028675-T01-IM6	PMS2 p.S547Rfs	55	IDC	NA	NA	4.3	NA	MSS	Neg	NA
breast_ink4_msk_2021	P-0030930-T01-IM6	MSH6 p.S156*	NA	NA	NA	M1	33.7	NA	MSS	Pos	NA
breast_ink4_msk_2021	P-0027617-T01-IM6	MLH1 p.X702_splice	NA	IDC	NA	M1	3.5	NA	MSI-H	Neg	NA
brca_igr_2015	MBC_149	MSH2 HOMDEL; MSH6 HOMDEL	NA	NA	HER2+	NA	0.1	NA	NA	Pos	NA
brca_igr_2015	MBC_157	PMS2 HOMDEL	NA	NA	NA	NA	0.3	NA	NA	Pos	NA
brca_igr_2015	MBC_180	PMS2 HOMDEL	NA	NA	NA	NA	2.1	NA	NA	Pos	NA
brca_igr_2015	MBC_182	PMS2 HOMDEL	NA	NA	NA	NA	5.3	NA	NA	Pos	NA
brca_igr_2015	MBC_82	MLH1 p.E277*	NA	NA	NA	NA	15.9	NA	NA	Neg	NA
brca_cptac_2020	X01BR040	PMS2 HOMDEL	NA	NA	TNBC	NA	1.4	NA	NA	Pos	NA
brca_cptac_2020	X11BR012	PMS2 HOMDEL	77	NA	HR+HER2-	NA	3.2	NA	NA	Pos	NA
brca_cptac_2020	X21BR001	MSH6 p.C1032*	NA	NA	HER2+	NA	8.7	NA	NA	Neg	NA

The asterisk (*) indicates a nonsense mutation (stop-gain). **Abbreviations:** MMR, Mismatch Repair; IDC, Invasive Ductal Carcinoma; ILC, Invasive Lobular Carcinoma; NA, Not Available or Not Applicable; HR, Hormone Receptor; HER2, Human Epidermal Growth Factor Receptor 2; TNBC, Triple-Negative Breast Cancer; TMB, Tumor Mutational Burden; MSI, Microsatellite Instability

Table S3. Comparison of the effects of various MMR mutation types on MMR function and immunogenicity in breast cancer

	CNV_Del	Frameshift Mutation	Nonsense Mutation	Splicing Mutation	Fusion	Missense Mutation	P-value
**No. of patients**	12	6	16	6	3	1	
**TMB (/Mb)**							**0.62**
Mean ± SD	5.9 ± 10.6	9.5 ± 10.8	23.6 ± 44.6	10.8 ± 14.3	1.8 ± 0.7	29.6	
Median (Range)	2.1 (0-37.9)	5.2 (0.7-28.7)	7.7 (0.3-142.4)	3.9 (0.1-35.1)	1.6 (1.1-2.6)	29.6	
**TMB class**							**0.481**
TMB-H	2 (16.7%)	2 (33.3%)	6 (37.5%)	2 (33.3%)	0 (0%)	1 (100%)	
TMB-L	10 (83.3%)	4 (66.7%)	10 (62.5%)	4 (66.7%)	3 (100%)	0 (0%)	
**Neoantigen (/Mb)**							**0.457**
Mean ± SD	16.5 ± 23.7	10.2 ± 15.1	64.3 ± 74.5	18.8 ± 7.6	2.6 ± 0.9	22.7	
Median (Range)	7.0 (0.4-51.8)	2.5 (0.6-27.6)	14.1 (8.0-158.8)	18.8 (13.4-24.1)	2.6 (1.8-3.5)	22.7	
**MSI status**							**0.06**
MSI-H	1 (20.0%)	0 (0%)	2 (28.6%)	3 (75%)	0 (0%)	1 (100%)	
MSS	4 (80.0%)	6 (100%)	5 (71.4%)	1 (25%)	3 (100%)	0 (0%)	
NA	7	0	9	2	0	0	
**MMRDetect**							**0.56**
Pos	1 (25.0%)	1 (33.3%)	1 (25.0%)	1 (50.0%)	0 (0%)	1 (100%)	
Neg	3 (75.0%)	2 (66.7%)	4 (75.0%)	1 (50.0%)	2 (100%)	0 (0%)	
NA	8	3	11	4	1	0	
**Mutation Sig6**							**0.24**
Mean ± SD	0.13 ± 0.18	0.09 ± 0.23	0.05 ± 0.18	0.19 ± 0.31	0 ± 0	0.14	
Median (Range)	0.1 (0-0.6)	0 (0-0.6)	0 (0-0.7)	0 (0-0.7)	0 (0-0)	0.1	

The P-value of TMB, Neoantigen and Mutation Sig6 was calculated using the Mann-Whitney U test. The P-value of TMB class, MSI status and MMRDetect was calculated using the Fisher exact test. **Abbreviations:** CNV, Copy Number Variation; Del, Deletion; TMB, Tumor Mutational Burden; MSI, Microsatellite Instability; Pos, Positive; Neg, Negative; Sig6, Signature6.

Table S4. Comparison of MMR function and immunogenicity between biallelic and monoallelic inactivation of MMR genes in breast cancers.

	MMR-altered	Mono	Bi	P-value
**No. of patients**	**44**	**23**	**21**	
**TMB (/Mb)**				**0.914**
Mean ± SD	13.8 ± 28.9	13.1 ± 29.3	15.9± 31.5	
Median (Range)	3.7 (0-142.4)	4 (0.1-128.4)	4.6 (0-142.4)	
**TMB class**				**0.744**
TMB-H	13 (29.5%)	6 (26.1%)	7(30.0%)	
TMB-L	31 (70.5%)	17 (73.9%)	14(70.0%)	
**Neoantigen (/Mb)**				**0.579**
Mean ± SD	27.0 ± 45.1	21.6 ± 39.8	33.9 ± 53.0	
Median (Range)	8.6 (0-158.8)	6.4 (0.6-131.6)	11.1 (0.4-158.8)	
**MSI status**				**0.095**
MSI-H	7 (27.0%)	2(13.3%)	5(50.0%)	
MSS	19 (73.0%)	13(86.7%)	6(50.0%)	
NA	18	8	10	
**Mutation Sig6**				**0.01**
Mean ± SD	0.11 ± 0.21	0.04 ± 0.13	0.17 ± 0.24	
Median (Range)	0 (0-0.7)	0 (0-0.6)	0 (0-0.7)	
**MMRDetect**				**0.619**
Pos	5 (29.4%)	2 (22.2%)	3 (37.5%)	
Neg	13 (70.6%)	7 (77.8%)	5 (62.5%)	
NA	26	14	14	

The P-value of TMB, Neoantigen and Mutation Sig6 was calculated using the Mann-Whitney U test. The P-value of TMB class, MSI status and MMRDetect was calculated using the Fisher exact test. **Abbreviations:** Mono, Monoallelic Inactivation; Bi, Biallelic Inactivation; TMB, Tumor Mutational Burden; MSI, Microsatellite Instability; Sig6, Signature6; Pos, Positive; Neg, Negative.

Figure S1. The positive control (PC) and negative control (NC) for MMR protein immunohistochemistry assays.

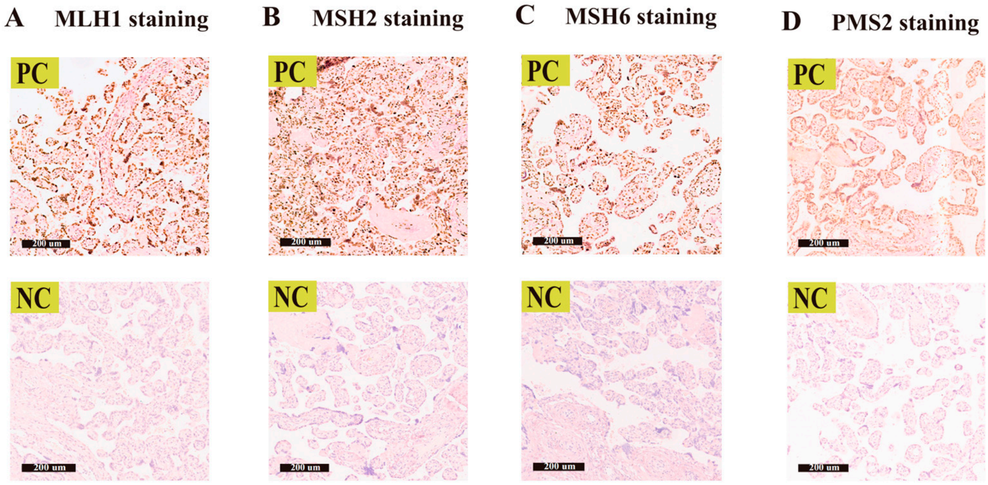

Supplement: Supplementary file 1 [file bioengineering-12-00426-s001.zip › bioengineering-3511082-supplementary.pdf]
